# A Novel Polyfunctional Polyurethane Acrylate Derived from Castor Oil-Based Polyols for Waterborne UV-Curable Coating Application

**DOI:** 10.3390/polym16070949

**Published:** 2024-03-30

**Authors:** Youmin Tuo, Xubiao Luo, Yahong Xiong, Chang-An Xu, Teng Yuan

**Affiliations:** Key Laboratory for Biobased Materials and Energy of Ministry of Education, College of Materials and Energy, South China Agricultural University, Guangzhou 510642, China

**Keywords:** waterborne polyurethane acrylate, comprehensive performance, castor oil, UV curing

## Abstract

Because of its unique molecular structure and renewable properties, vegetable oil has gradually become the focus of researchers. In this work, castor oil was first transformed into a castor oil-based triacrylate structure (MACOG) using two steps of chemical modification, then it was prepared into castor oil-based waterborne polyurethane acrylate emulsion, and finally, a series of coating materials were prepared under UV curing. The results showed that with the increase in MACOG content, the glass transition temperature of the sample was increased from 20.3 °C to 46.6 °C, and the water contact angle of its surface was increased from 73.85 °C to 90.57 °C. In addition, the thermal decomposition temperature, mechanical strength, and water resistance of the samples were also greatly improved. This study not only provides a new idea for the preparation of waterborne polyurethane coatings with excellent comprehensive properties but also expands the application of biomass material castor oil in the field of coating.

## 1. Introduction

Polyurethane acrylate is a kind of cross-linked polymer with a carbamate structure that is synthesized by a polyaddition reaction of polyol and isocyanate. It is widely used in coatings, leather, adhesives, and sealants because of its excellent adhesion, flexibility, wear resistance, and weather resistance [1,2]. However, the viscosity of traditional polyurethane acrylate is relatively high, and active diluents are usually added to regulate it. The active diluents commonly used are mainly acrylates with low boiling point, volatile, irritating odor, and toxicity, which will bring great harm to the environment and human health. With the increase in people’s awareness of environmental protection and the implementation of environmental protection policies, people have gradually turned their attention to pollution-free and green water. Its use as an active diluent in the polymerization process can not only reduce the viscosity of the polymer but also reduce the release of VOC [3,4,5,6,7] and enhance operational safety during use. In addition, the coating industry has begun to transform towards environmentally friendly products. Compared with traditional organic coatings, UV-cured coatings have advantages such as low VOC emissions, high curing efficiency, convenient operation, pollution-free properties, and low energy consumption [8,9,10,11]. In the field of photochemistry, UV curing has maintained an unprecedented position in the new generation of industry-related coating systems, and therefore, UV curing coatings have become one of the research hotspots.

With the rapid development of modern society, due to the increasing consumption of petrochemical products, resulting in the increasing shortage of petroleum raw materials, people gradually turn their attention to rich resources and low-cost, renewable, and biodegradable biomass materials [12,13,14,15,16,17,18]. Among numerous biomass materials, vegetable oil has received extensive attention from researchers due to its unique molecular structure, abundant content, suitable price, and biodegradability [19,20]. A variety of modified vegetable oil products can be prepared by using active sites such as C=C double bond, hydroxyl group, epoxy group, and ester group on vegetable oil, such as polyol materials for the synthesis of polyurethane acrylate [21,22]. For example, Wang et al. synthesized an oleic acid-based primary alcohol using methyl oleate as raw materials, which exhibited high reactivity as a polyurethane soft segment. At the same time, the prepared film material showed excellent tensile properties [23]. Li et al. synthesized epoxy soybean oil acrylate with multi-functional hydroxyl groups from epoxy soybean oil and introduced it into the main chain of waterborne polyurethane acrylate [24]. The results showed that the epoxy soybean oil-based solidified film had good mechanical and thermodynamic properties, and its material had been well used in the field of wood coatings. Moreover, Gaddam et al. used cottonseed oil to synthesize three kinds of phosphorylated polyols with different hydroxyl values and used them as soft segments of polyurethane to synthesize waterborne polyurethane dispersions (PUDs) without industrial hydrophilic chain extension and catalyst. The three types of PUDs exhibited excellent storage stability, and the tensile properties, glass transition temperature, thermal stability, hydrophobicity, and anti-corrosion properties of the coating all improved with the increase of the hydroxyl value of the phosphorylated polyols [25]. It can be concluded that the introduction of vegetable oil and its derivatives can effectively improve the mechanical properties and thermal stability of waterborne polyurethane acrylate coatings. However, few studies have reported that vegetable oil is used both as a raw material for polyurethane reaction and as a source of UV-curable double bond monomer.

In this study, castor oil was chemically modified by a two-step method with maleic anhydride, a biomass resource, to prepare multi-functional castor oil triacrylate (MACOG), and then different percentages of MACOG were introduced into waterborne polyurethane acrylate emulsion, and a series of coating materials were prepared by UV curing [26]. Subsequently, the thermodynamic properties, mechanical properties, thermal stability, gel content, and hydrophobic properties of the coating were tested. It was concluded that the vegetable oil derivatives could be used to prepare high-performance coating materials instead of petrochemical resources. Most importantly, this work not only made the raw materials sustainable but also made the preparation and curing processes green and pollution-free.

## 2. Experiment

### 2.1. Materials

Castor oil (CO) was sourced from Tianjin Fuyu Fine Chemical Reagent Co., Ltd. (Tianjin, China). Dibutyltin dilaurate (DBTDL) and maleic anhydride (MA) were purchased from Tianjin Fuchen Chemical Reagent Co., Ltd. (Tianjin, China). Isophorone diisocyanate (IPDI), glycidyl methacrylate (GMA), polybutanediol adipate (PBA, 1000 g/mol), hydroxyethyl acrylate (HEA), and dimethylol butyric acid (DMBA) were all derived from Shanghai Maclin Biochemical Co., Ltd. (Shanghai, China). Both hydroquinone and N, N-dimethylethanolamine (DMEA) were from Tianjin Damao Chemical Reagent Co., Ltd. (Tianjin, China). Triethylamine (TEA) was from Tianjin Yongda Chemical Reagent Co., Ltd. (Tianjin, China). 2-butyl ketone was purchased from Guangzhou Chemical Reagent Factory. 2-hydroxy-2-methyl-1-phenylacetone (PI-1173) was purchased from Tianjin Jiuri New Materials Co., Ltd. (Tianjin, China). The above pharmaceutical materials had not been further processed and had been directly used. The UV equipment (CH-UV06) used was from Shanghai Yuming Instrument Co., Ltd., Shanghai, China. The UV lamp had a power of 85 W and a wavelength of 395 nm.

### 2.2. Synthesis of Castor Oil with Ternary Carboxylic Acid

**Scheme 1:**
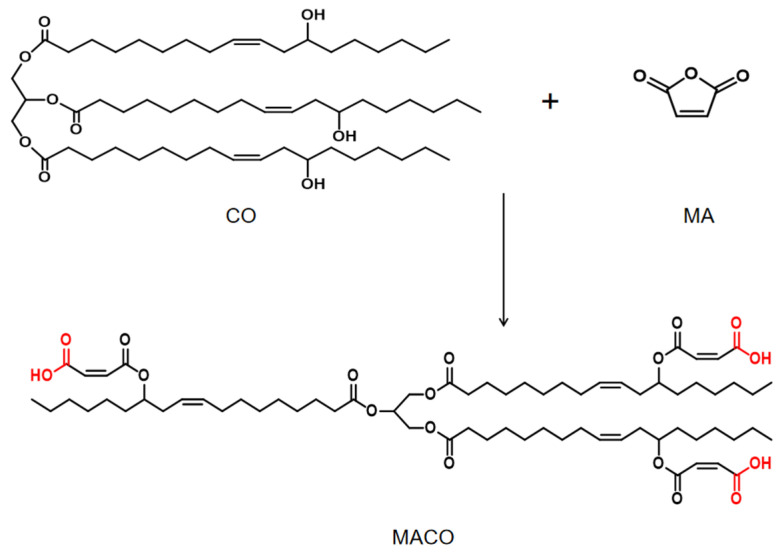
The process of preparing MACO.

A total of 34.21 g of castor oil, 9.81 g of maleic anhydride, 0.09 g of hydroquinone, and 0.22 g of DMEA were added to the three-neck round bottling flask. The reaction mixture was then heated to 65 °C and stirred at 250 r/min until the MA was completely melted. The mixture was then heated to 105 °C and reacted for 3 h to obtain a yellow, transparent, and viscous liquid of castor oil-based terarboxylic acid, which was named MACO. The synthesis process is shown in Scheme 1.

### 2.3. Synthesis of Castor Oil-Based Triacrylate

**Scheme 2:**
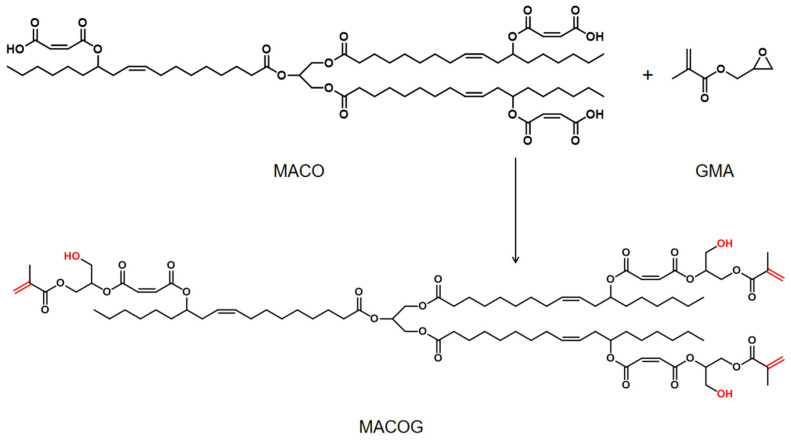
The process of preparing MACOG.

A total of 44.02 g of MACO and 0.29 g of DMEA were added to a 250 mL three-necked round-bottomed flask and heated to 90 °C. Then, the mixture of 14.66 g GMA and 0.15 g hydroquinone was added to the mixture by drops within 30 min and reacted for 4 h. Finally, a slightly orange viscous liquid of castor oil-based triacrylate was obtained and named MACOG. The synthesis route is shown in Scheme 2.

### 2.4. Synthesis of Waterborne Polyurethane Acrylate Emulsion (WPUA)

**Scheme 3:**
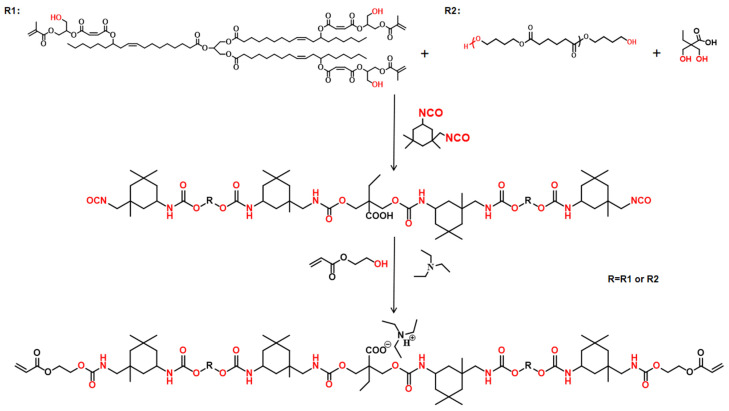
The process of preparing WPUA.

First, appropriate amounts of DMBA, PBA, and IPDI were added to a 250 mL three-necked round-bottomed flask. Two drops of DBTDL and appropriate amounts of 2-butanone were added to the mixture. The mixture was stirred at 80 °C for 3 h in a nitrogen atmosphere. Subsequently, MACOG with different mass fractions was added to the mixture by drops within 30 min, and the detailed formula is shown in Table 1. After 4 h of reaction, HEA containing 1 wt% hydroquinone was added to the flask. When NCO was completely consumed, the mixture was cooled to room temperature, and an appropriate amount of TEA was added and then stirred for 30 min to neutralize the carboxyl group. Finally, 30 wt% deionized water was added and stirred at high speed for 2 h, and 2-butanone was removed to obtain waterborne polyurethane acrylate dispersion (WPUA). The synthesis route is shown in Scheme 3.

### 2.5. Preparation of Waterborne Polyurethane Acrylate Photocurable Film

A certain amount of WPUA lotion and 4 wt% of PI-1173 were added into a 20 mL glass bottle and stirred evenly to form an aqueous dispersion. Subsequently, the dispersion was poured into a culture dish and dried at room temperature for 24 h before drying at 60 °C for 12 h. Finally, the cured film was obtained by irradiation for 30 s under a UV lamp with a distance of 2 cm and a light intensity of 387 mW/cm^2^. The thickness of the obtained film was 0.4 mm.

### 2.6. Characterization

The liquid samples CO, MACO, and MACOG were coated on halide chips to form a liquid film and characterized using Fourier transform infrared spectroscopy (FTIR, Nicolet iS10, Thermo Fisher, Waltham, MA, USA), with a wavelength range of 500–4000 cm^−1^. The proton shift of the product was characterized using a nuclear magnetic resonance spectrometer (Bruker AV 400, Bruker Biospin AG, Fällanden, Switzerland), and the solvent used in the test was CDCl_3_. The dynamic mechanics of the sample were tested using the tensile mode of a dynamic thermomechanical analyzer (DMA 242E, German Naichi, Krefeld, Germany). The size of the test sample was 20.0 mm × 6.0 mm × 0.5 mm, with an oscillation frequency of 1 Hz, a testing temperature of −80~180 °C, and a heating rate of 3 K/min. Formula (1) was used to calculate the cross-linking density of the sample.

V_e_ = E’/3RT’, (1)

where T’ is the absolute temperature (T_g_ + 30 °C) in the rubber state, E’ is the storage modulus at T’, and R is the gas constant 8.314 J/(mol·K).

An electronic universal testing machine (UTM4204, Shenzhen Sansi Zongheng Technology Co., Ltd., Shenzhen, China) was used to test the tensile properties of the cured film according to the GB/T 1040.2-2006 standard. The tensile speed was set at 20 mm/min, and the sample size was 40.0 mm × 10.0 mm × 0.5 mm. The thermal stability of the sample in the nitrogen atmosphere was tested using a thermal analyzer (TG209F1LibraTM, Germany Nechi instrument manufacturing Co., Ltd. Shanghai, China). The heating rate of the test was 10 °C/min, and the temperature range was 35~800 °C. The particle size distribution and Zeta potential were measured using a laser particle size analyzer (Zetasizer Nano ZSE, Malvern, Shanghai, China). Before testing, the sample was diluted 100 times with distilled water. According to the GB/T 30693-2014 standard, a contact angle measuring instrument (OCA20, DATAPHYSICS, Shanghai, China) was used to test the water contact angle of the cured film surface. The mass of the sample before testing was m_0_. The sample was taken out after soaking in water for 48 h, and the surface moisture was absorbed with filter paper. The mass of the sample was denoted as m_1_. This process was repeated three times, and its average was finally taken. According to Formula (2), the water absorption rate of the solidified film was calculated.

Water absorption rate = (m_1_ − m_0_)/m_0_ × 100% (2)

The acetone extraction method was used to test the gel content of the solidified film. The steps were as follows: First, the solidified film with a mass of W_0_ was accurately weighed at room temperature and then soaked in a sealed glass bottle containing acetone for 48 h. After that, the solidified film was taken out and dried in a vacuum oven at 60 °C to a constant weight, and its weight was weighed as W_1_. The gel ratio was calculated according to Formula (3).

Gel ratio = (W_1_/W_0_) × 100% (3)

The flexibility of the coating was tested according to GB/T 1731-1993. The pencil hardness of the coating was tested according to GB/T 6739-1996. According to GB/T 9274-1988, the acid and alkali resistance of the coating was tested.

## 3. Results and Discussion

### 3.1. Structural Characterization of Products

**Figure 1:**
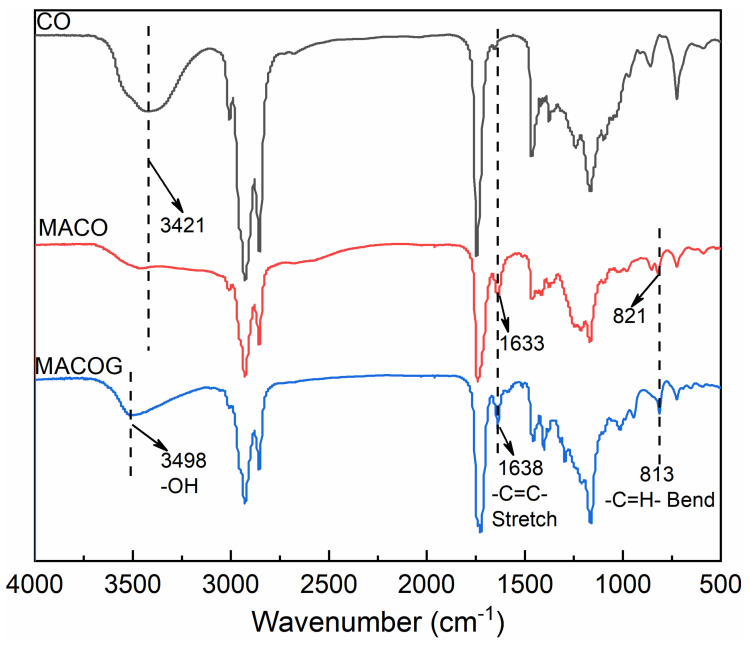
Infrared spectra of CO, MACO and MACOG.

**Figure 2:**
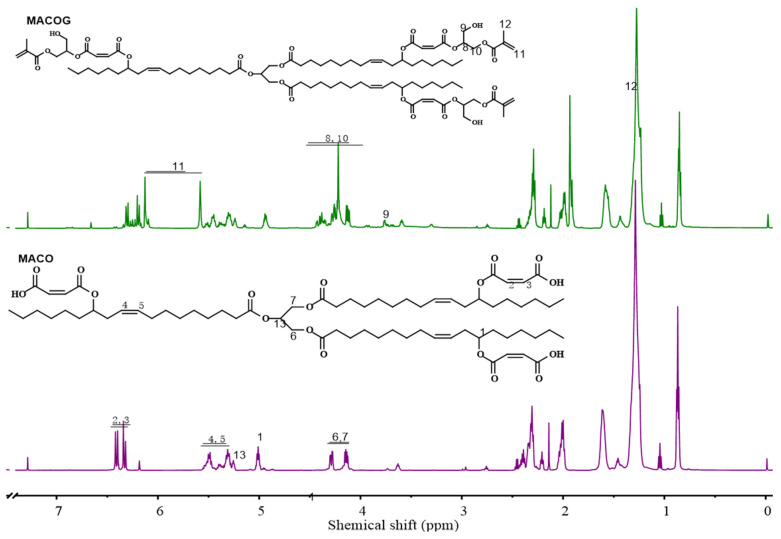
^1^H-NMR spectra of MACO and MACOG.

The infrared spectra of CO, MACO, and MACOG are shown in Figure 1. It could be seen that the characteristic hydroxyl peak of CO was found at 3421 cm^−1^. However, in MACO, the characteristic peak at 3421 cm^−1^ disappeared, while a wide and strong carboxyl absorption peak appeared near 2500–3500 cm^−1^ [27]. This indicated that MACO had been successfully synthesized through the esterification reaction between CO and MA. In the infrared spectrum of MACOG, a new hydroxyl peak appeared at 3498 cm^−1^, and the characteristic peaks at 1638 cm^−1^ and 813 cm^−1^ were attributed to the C=C stretching vibration absorption peak and the C=H bending vibration absorption peak, respectively. This indicated that MACOG had been successfully synthesized through the ring-opening esterification reaction between MACO and GMA. Subsequently, nuclear magnetic tests were used to further confirm that MACO and MACOG had been successfully synthesized, and the results are shown in Figure 2. MACO spectra showed that the hydroxyl proton peak disappeared at 3.63 ppm in CO, and a new chemical shift peak appeared at 5.00 ppm, which corresponded to the proton peak of newly formed ester-linked methylene [28]. In the MACOG spectra, the chemical shift at 3.82–4.54 ppm should be attributed to the methylene and methylene proton peaks in the structure of methacrylate. The chemical shift of the -CH_2_- proton peak attached to the newly formed hydroxy-group appeared at 3.76 ppm, while the chemical shift of the -CH=CH_2_ structure in GMA appeared at 5.57–6.18 ppm.

### 3.2. Particle Size Analysis of Emulsion

**Figure 3:**
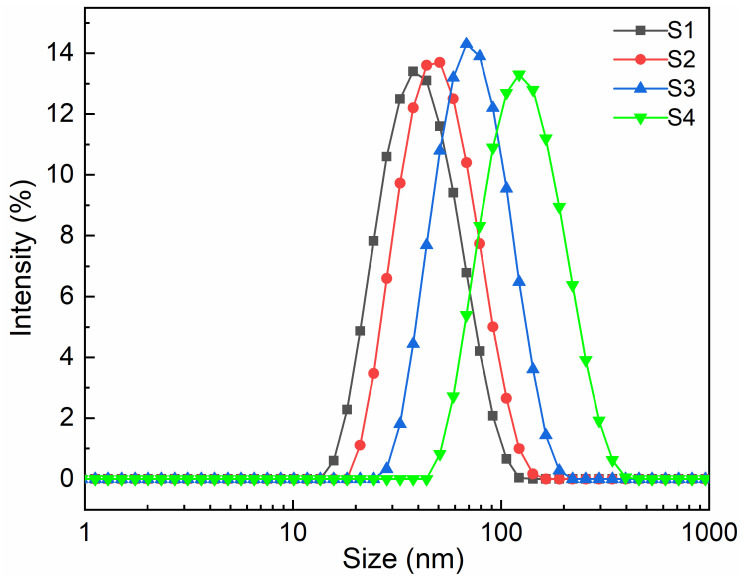
Particle size distribution curves of emulsion with different mass fractions of MACOG.

The storage stability of emulsion could be determined by measuring emulsion particle size distribution and Zeta potential [29]. The test results of the samples are shown in Figure 3 and Table 2. As can be seen from Figure 3, with the increase in MACOG content, the mean particle size (From 36.87 nm to 118.60 nm) and particle size distribution (From 0.136 to 0.165) of emulsion showed a trend of increasing and widening, respectively. This was mainly attributed to the increase in MACOG content, which reduced the relative content of DMBA in raw materials. After neutralization, the number of ionic groups was reduced, which would weaken the electrostatic repulsion between dispersed particles and improve the association of particles. In addition, the increase in MACOG would also introduce more hydrophobic chain segments, which would not only enhance the hydrophobicity of oligomers but also improve the cross-linking degree of prepolymers (as seen in Table 3). It can be seen from Table 2 that the PDI values of all emulsions were less than 0.3, indicating that they all had good dispersion. There was no obvious precipitation after 6 months at room temperature, and the Zeta potential was higher than 42.0 mV, indicating that the WPUA emulsion prepared in this work had good storage stability.

### 3.3. Dynamic Thermomechanical Properties

**Figure 4:**
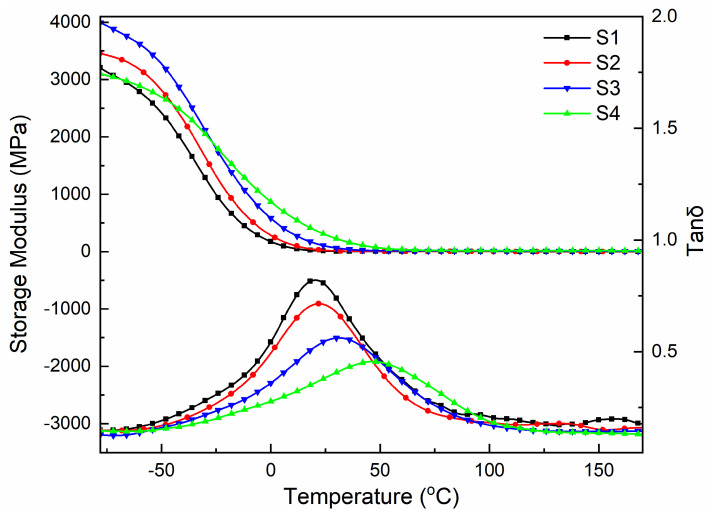
DMA curves of cured films with different MACOG content.

The storage modulus (E’), glass transition temperature (T_g_), and loss factor (Tanδ) of the cured film obtained through dynamic mechanical analysis are shown in Figure 4 and Table 3. As can be seen from Figure 4, the cured film exhibited a high storage modulus in the low-temperature region, and its value first increased and then decreased with the increase in MACOG content. At the same time, the storage modulus of all cured films was temperature-dependent, and its value decreased with the increase in heating temperature. The samples all showed a peak of the T_g_ on the Tan δ curve, which indicated that the cured film was homogeneous and the compatibility between the substances was good. At the same time, the peak shape decreased and widened with the increase in MACOG content, which was mainly related to the increase in the cross-linking density of the cured film [30]. The higher the cross-linking density, the weaker the mobility of the chain segment and the higher the glass transition temperature. As can be seen from Table 3, with the increase in MACOG content, the cross-linking density of samples increased, which was mainly related to the increased double-bond content in the system. The increase in cross-linking density would weaken the kinematic ability of chain segments in the system and then increase the T_g_ of the cured film, resulting in the T_g_ of the system increased from 20.3 °C to 46.6 °C.

### 3.4. Mechanical Property

**Figure 5:**
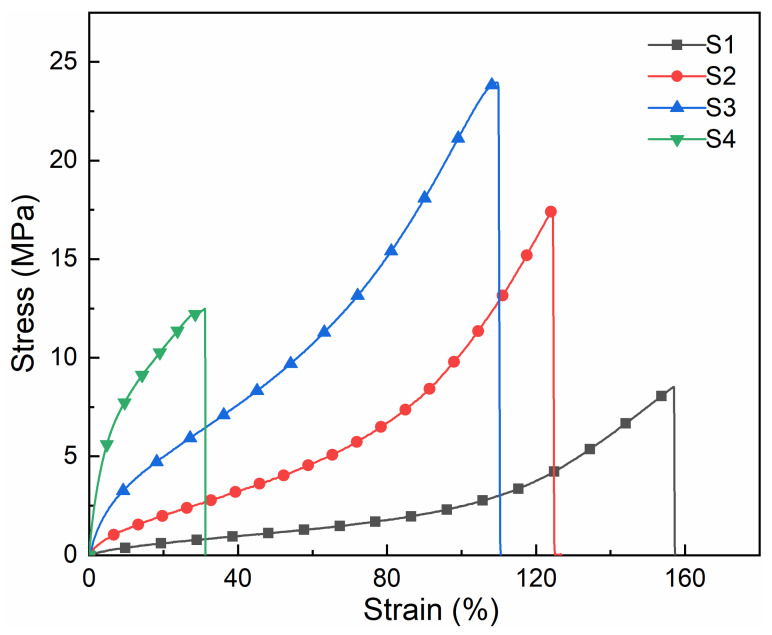
Stress-strain curves of cured films with different MACOG content.

The test curve of the mechanical properties of the cured film is shown in Figure 5. It could be concluded that with the increase in MACOG content, the fracture strength and Young’s modulus of the cured film showed a trend of first increasing and then decreasing. The increase in fracture strength of cured film was mainly related to the increase in cross-linking density in the system (As shown in Table 3). However, when the content of MACOG reached 40 wt%, the fracture strength and Young’s modulus of the sample were reduced. This was mainly because excessive cross-linking density in the system would not only lead to poor compatibility between hard and soft segments in the system but also cause an uneven distribution of cross-linking sites in the system [31]. When the content of MACOG was 30 wt%, the sample S3 had the largest breaking strength, which was about 23 MPa.

### 3.5. Thermal Stability

**Figure 6:**
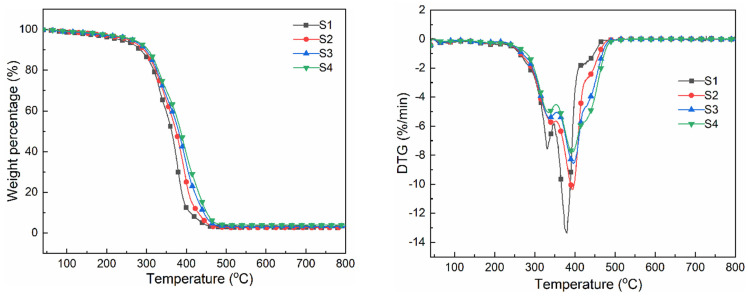
TGA and DTG curves of cured films with different MACOG content.

The thermogravimetric method was used to analyze the thermal stability of the cured film, and its TGA and DTG curves are shown in Figure 6. It can be seen from the thermal decomposition curve that the cured film experienced three thermal degradation stages, and the degradation below 200 °C was mainly related to the residual moisture in the cured film. The degradation at 230 °C to 360 °C was mainly related to the decomposition of carbamate [32]. With the increase in MACOG content, the unstable content of carbamate in the system was reduced so that the thermal decomposition temperature was increased [33]. The decomposition between 360 °C and 500 °C was mainly related to the fracture of soft segments and cross-linking bonds in polyurethane. The temperature corresponding to a sample mass loss of 5 wt% (T_5%_) is generally used as the starting decomposition temperature of the sample. From Table 4, it could be seen that the temperature of sample S1 at T_5%_ was 231.9 °C. With the increase in MACOG content, the T_5%_ value of the sample further increased, reaching a maximum of 264.7 °C. In addition, when the mass loss of the sample was 30 wt% (T_30%_), the T_30%_ value of sample S1 was still the smallest, and its trend of change was consistent with T_5%_. After all samples were heated at high temperatures, the residual char rates of samples S1, S2, S3, and S4 at 790 °C were 2.5 wt%, 2.6 wt%, 3.2 wt%, and 3.8 wt%, respectively. This indicated that the residual char rate of samples at high temperatures increased with the increase in MACOG content. All samples in the DTG curve exhibited two temperature peaks corresponding to the maximum thermal weight loss rate, which was caused by the thermodynamic incompatibility between the soft and hard segments of polyurethane. The thermal decomposition temperature of the hard segment was lower than that of the soft segment, so the maximum thermal decomposition rate at low temperatures was mainly related to the degradation of the hard segments. In addition, as MACOG increased, the cross-linking density of the sample increased, causing the maximum thermal weight loss rate temperature peak of the sample to shift toward higher temperatures. It could be concluded that the introduction of MACOG would increase the cross-linking density of the system, thereby improving the thermal stability of the system.

### 3.6. Contact Angle and Water Absorption

**Figure 7:**
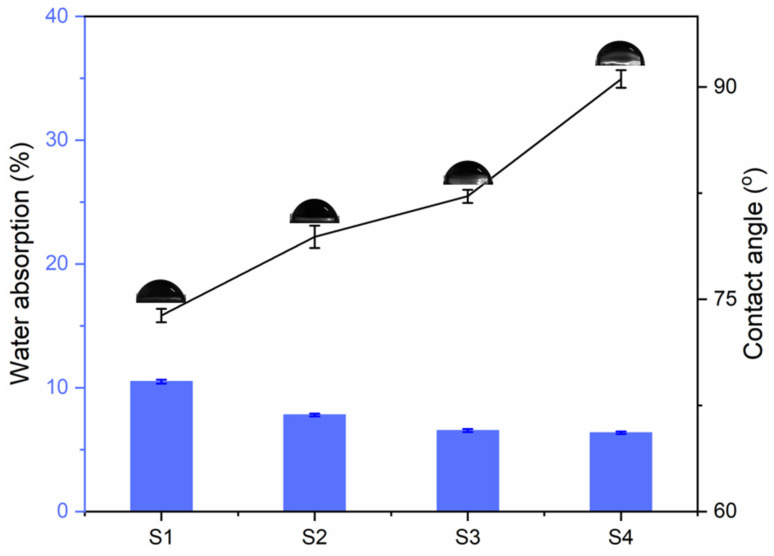
Water absorption and water contact angle of cured films with different MACOG contents.

The test results of water absorption and surface water contact angle of the cured coating are shown in Figure 7. With the increase in MACOG content, the water absorption of the cured film decreased from 10.49 wt% to 6.36 wt%, and the water contact angle increased from 73.85° to 90.57°, which indicated that the introduction of MACOG could effectively improve the water-resistance of the cured film. On the one hand, it was related to the structure of hydrophobic long-chain fatty acids in MACOG [34]. On the other hand, it was related to the increased cross-linking density in the system, which enhanced the compactness of the coating [35].

### 3.7. General Performance of Curing Film

The general performance tests of the cured film are shown in Table 5. With the increase of the content of MACOG, the gel content and pencil hardness of the cured film showed the same trend of change. The gel ratio increased from 78.44 wt% to 94.71 wt%, and the pencil hardness increased from HB to 2H, which was mainly related to the increase in the cross-linking density of the system caused by the increase in MACOG. All cured films exhibited excellent flexibility, mainly due to the long-chain structure of PBA and the fatty acid long-chain structure of MACOG. By testing the acid and alkali resistance of the cured film, it was found that there was no significant change after immersion in HCl aqueous solution, indicating good acid resistance. However, after soaking in a NaOH solution, the surface of the cured film appeared white, which was mainly because the ester group contained in the structure of polyurethane was easy to hydrolysis in NaOH, leading to the destruction of its structure and weakening its alkali resistance [36].

## 4. Conclusions

In this work, a new type of multi-functional castor oil-based acrylate (MACOG) was successfully prepared using castor oil, maleic anhydride and glycidyl methacrylate as raw materials, and then it was synthesized into waterborne polyurethane acrylate emulsion, a variety of coating materials were prepared under UV, and the comprehensive performance of the cured film was evaluated. The results showed that the introduction of MACOG could effectively improve the thermal stability, glass transition temperature, acid resistance, gel rate, and breaking strength of cured films. The water contact angle of the coating surface increased from 73.85° to 90.57°, while the water absorption decreased from 10.49 wt% to 6.36 wt%. This was mainly related to the introduction of MACOG, which improved the cross-linking density in the system. In conclusion, this work not only prepared coatings with excellent comprehensive properties but also improved the added value of castor oil, which provided a new research idea for using vegetable oil instead of petrochemical resources to prepare coating products.

## Figures and Tables

**Table 1:** Formula of WPUA.

Samples	Mass Fraction of MACOG (wt%)	Raw Material Formula (g)					
		IPDI	PBA	MACOG	DMBA	HEA	TEA
S1	10	4.82	10	1.84	1.14	0.6	0.78
S2	20	5.30	10	4.72	1.14	0.6	0.78
S3	30	5.97	10	7.60	1.14	0.6	0.78
S4	40	6.95	10	12.47	1.14	0.6	0.78

**Table 2:** Average particle size, PDI value, and Zeta potential of WPUA with different MACOG content.

Samples	DMBA Content (wt%)	Mean Particle Size (nm)	Dispersion Coefficient (PDI)	Zeta Potential (mV)
S1	6.20	36.87 ± 0.17	0.136 ± 0.009	44.43 ± 2.32
S2	5.24	45.63 ± 0.27	0.134 ± 0.010	42.73 ± 1.27
S3	4.50	66.37 ± 0.19	0.138 ± 0.004	45.30 ± 1.24
S4	3.66	118.60 ± 0.08	0.165 ± 0.003	55.17 ± 1.69

**Table 3:** DMA data and cross-linking density of cured films with different MACOG content.

Samples	E_25_ (MPa)	T_g_ (°C)	E’ at T_g_ + 30 °C (MPa)	υ_e_ (×10^3^ mol/m^3^)
S1	9.21	20.3	2.82	0.35
S2	22.89	22.1	6.39	0.79
S3	99.08	31.2	13.08	1.57
S4	298.59	46.6	23.74	2.72

**Table 4:** Thermogravimetric data of cured films with different MACOG content.

Samples	T_5%_ (°C)	T_30%_ (°C)	Carbon Residue Rate (790 °C, wt%)
S1	231.9	332.9	2.5
S2	253.9	340.9	2.6
S3	262.0	344.5	3.2
S4	264.7	349.6	3.8

**Table 5:** General properties of cured films with different MACOG contents.

Classification	S1	S2	S3	S4
Gel rate (wt%)	78.44	87.27	91.88	94.71
Pencil hardness	HB	H	2H	2H
Flexibility (mm)	2	2	2	2
Acid resistance (0.1 mol/L HCl)	Unchanged	Unchanged	Unchanged	Unchanged
Alkali resistance (0.1 mol/L NaOH)	Bleach	Bleach	Bleach	Bleach

## Data Availability

Data are contained within the article.
